# A Multi-level Perspective on the Evolution of Orthologs and Their Functions

**DOI:** 10.1007/s00239-025-10276-2

**Published:** 2025-10-13

**Authors:** Felix Langschied, Ruben Iruegas, Mateusz Sikora, Roberto Covino, Ingo Ebersberger

**Affiliations:** 1 Institute of Cell Biology and Neuroscience, Applied Bioinformatics Group, Goethe University Frankfurt, Frankfurt am Main, Germany; 2 Malopolska Centre of Biotechnology, Jagiellonian University, Krakow, Poland; 3 Department of Theoretical Biophysics, Max Planck Institute of Biophysics, Frankfurt am Main, Germany; 4 Institute of Computer Science, Goethe University Frankfurt, Frankfurt am Main, Germany; 5 Frankfurt Institute for Advanced Studies (FIAS), Frankfurt Am Main, Germany; 6 Senckenberg Biodiversity and Climate Research Centre (S-BIK-F), Frankfurt am Main, Germany; 7 LOEWE Centre for Translational Biodiversity Genomics (TBG), Frankfurt am Main, Germany

**Keywords:** Protein activity, Annotation transfer, Feature architecture, Protein domain, Homology, Metabolic pathway

## Abstract

Orthologs, evolutionarily related genes that diverged through speciation, are mutually the closest related sequences in different species. Consequently, they are ideal candidates for identifying functionally equivalent genes across taxa, a prerequisite for transferring gene function information from model to non-model organisms in silico. However, orthologs are not immune to functional divergence. Failing to recognize such divergent instances results in spurious functional annotation transfer. Here, we propose to treat the functional equivalence of orthologs as a null hypothesis that must be critically tested rather than assumed. This requires integrating several lines of evidence to evaluate both changes in an ortholog’s network of molecular interactions and alterations in its biochemical activity. We outline how such activity shifts can be assessed using increasingly fine-grained analyses, including comparisons of protein feature architectures and predicted 3D structures. While some orthology resources incorporate aspects of this evidence, such assessments are often manual and not scalable. We argue for a systematic, multi-level perspective to detect functional divergence prior to annotation transfer. To support the broader adoption of this approach, we offer methodological recommendations and practical examples that demonstrate the value of this framework in large-scale comparative genomics.

## Introduction

One of the central challenges in biology is linking an organism’s phenotype to its genotype. Experimental assays—such as gene knockouts—offer direct evidence of which genes influence specific phenotypes within individual species (Giaever and Nislow 2014). However, these approaches are time-consuming, costly, and typically limited to a small number of model organisms (Irion and Nüsslein-Volhard 2022). As a result, despite the exponentially growing number of available genome sequences that increasingly represent the phenotypic diversity of cellular life (Arita et al. 2021; Lewin et al. 2022), most newly sequenced genes remain experimentally uncharacterized (de Crécy-Lagard et al. 2022).

Since the advent of the genomics era in the early 2000s, comparative genomics has become a powerful approach for extending genotype–phenotype associations from model organisms to non-model species (Koonin 2005). Once a gene has been experimentally characterized in a model species, the task is to identify the “corresponding” gene in another species. This should be all genes with shared evolutionary ancestry, also known as homologs, under the condition that all homologs maintained their ancestral association to the phenotype. However, paralogs (homologs stemming from gene duplication) are typically assumed to diversify quickly, since mutations can accumulate more freely in redundant genes. As a consequence, the redundant copy often becomes dysfunctional, or the paralogs either subfunctionalize or one copy acquires a novel function (neofunctionalization) (e.g., Ohno 1970; Taylor and Raes 2004; Kuzmin et al. 2022).

In contrast, orthologs (homologs split by speciation events) evolve, at least initially, under purifying selection that maintains the ancestral gene function. Hence, identifying orthologs has become a routine strategy for transferring functional knowledge across species (Mi et al. 2010). A wide array of orthology inference methods is now available, each balancing trade-offs between sensitivity, specificity, and scalability (Altenhoff et al. 2024a, b). Consequently, the presence or absence of orthologs, also known as a phylogenetic profile, can be efficiently inferred for any gene of interest across large, taxonomically diverse species sets that increasingly span the tree of life (Kuznetsov et al. 2023; Persson and Sonnhammer 2023; Altenhoff, Warwick Vesztrocy, et al. 2024; Tran et al. 2024).

The interpretation of phylogenetic profiles in the context of functional evolution seems, at first sight, straightforward. A missing ortholog in a phylogenetic profile is interpreted as a loss of the gene’s function—provided the ortholog is truly absent from the species’ genome and not simply undetected (Jain et al. 2019; Weisman et al. 2020). This inference is further supported by the rarity of non-homologous replacements, where a protein responsible for a specific phenotype in one species is functionally substituted by an unrelated protein in another without an observable phenotypic change (Omelchenko et al. 2010). Conversely, the presence of an ortholog in a species is often taken as evidence for the presence of the corresponding function. This assumption is frequently justified by citing the ortholog conjecture (Gabaldón and Koonin 2013) and is often accepted without further validation. However, the conjecture states that ‘functionally equivalent proteins in two species are most likely orthologs’—not that all orthologs necessarily perform equivalent functions.

Meanwhile, there is ample evidence that orthologs can indeed functionally diversify and contribute to varying phenotypes in different species (Dey and Meyer 2015). For example, replacing essential yeast genes with their 1:1 human ortholog produced viable cells in 40% of cases (Laurent et al. 2020). Given that the two species last shared a common ancestor more than 1 billion years ago (Kumar et al. 2022), this number is surprisingly high, and it underlines that orthologs are at least a good guess when looking for functionally equivalent proteins in two species. However, at the same time, it demonstrates that the majority of orthologs have functionally diversified since then. Therefore, the commonly held view that 'orthologs perform the same function across different species' is overly simplistic.

**Fig. 1:**
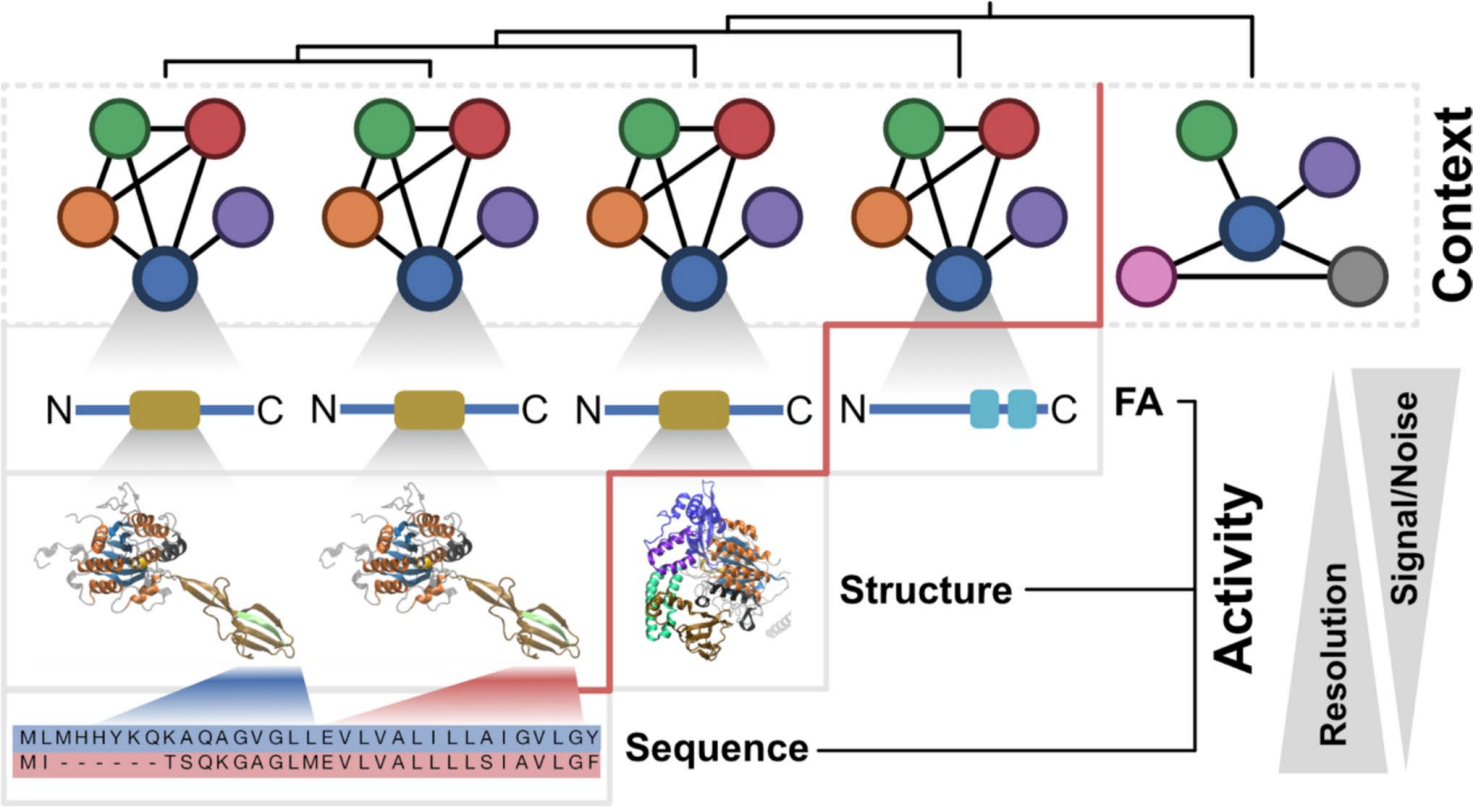
Multi-level perspective for identifying the diversification of orthologs. The top row (grey dotted box) shows orthologs across five species (ordered by increasing evolutionary distance from left to right) as colored circles. Black lines indicate connections between the focal ortholog (blue) and its interaction partners, forming its functional context. Below, increasingly fine-grained levels of comparative analyses to detect divergence of biochemical activity are shown in grey boxes: feature architecture (FA), 3D-structure of the brown protein domain highlighted on FA level, and the sequence alignments corresponding the finger-like structure of the left-most ortholog (blue) and the adjacent one (red) (from (Iruegas et al. 2023)). A red vertical line marks the level at which functional divergence between orthologs is detected. The trade-off between a higher resolution and the signal-to-noise ratio is depicted with two triangles

A more nuanced understanding of the relationship between orthology and functional equivalence—or divergence—is essential for mitigating the impact of spurious functional annotations on the quality of protein databases (Gilks et al. 2002; Bell et al. 2013). Conceptually, functional diversification can be attributed to two different processes that act on different scales: the change of a gene’s “biochemical activity”, and the change of its “functional context” (Fig. 1). A gene's *biochemical activity* refers to its causal effects, such as the ability of the encoded protein to bind specific molecules or to catalyze a certain biochemical reaction (Doolittle et al. 2014). A change in *biochemical activity* is, therefore, always connected to a change of the gene and of the encoded protein itself, for example, by the gain, loss, or modification of catalytic or binding domains. The often-used term *function* should be reserved for explaining why a feature persists (Doolittle et al. 2014). More precisely, *function* can be defined as features of a biological entity that contributed to its survival over evolutionary time scales (Griffiths 2009; Graur et al. 2013). To describe a gene in a more holistic way, the ‘functional annotation’ of a gene should, therefore, not only comprise its biochemical activity, but also its ‘functional context’, e.g., the overarching processes in which a gene’s biochemical activity is embedded, such as a metabolic pathway or a multi-protein complex.

To give one example of how frameworks for standardizing functional annotations are capturing this relationship: the Gene Ontology (GO) records *biochemical activity* as a gene’s “Molecular Function”. This activity can then be contributed to different “Biological Processes” and can be localized in different “Cellular Components” (Ashburner et al. 2000). Integrating annotation from all three categories informs then about the *function* of a gene as it was described above. Thus, the GO already captures that the same gene can contribute to different functions in two species, for example, by acquiring different interaction partners even if its biochemical activity remains unchanged.

At first glance, the distinction between a gene’s biochemical activity and its functional context may seem artificial, as changes in either can impact the phenotype. However, in the context of the ortholog conjecture, this distinction becomes important, because it determines the criteria that must be met to justify the transfer of functional annotations between orthologs. To motivate this, we briefly recapitulate the conceptual idea that underlies the ortholog conjecture. It is fair to assume that any two orthologous genes in two very recently diverged species, e.g., humans and chimpanzees, have both the same biochemical activity and the same functional context. This is because of their shared ancestry and because not enough time has passed for divergence (Ebersberger et al. 2002). From this follows that the equivalence of biochemical activity and functional context of orthologs is the null hypothesis. Consequently, an approach that uses orthology relationships in the context of an annotation transfer must challenge this hypothesis to faithfully differentiate between those orthologs that have most likely experienced changes in biochemical activity and/or functional context, and those for which no such evidence exists. Although orthology is a concept that applies to the gene level, biochemical activity and functional context are best described and investigated on the level of the encoded proteins. We will, therefore, use *gene* and *protein* interchangeably in the following sections, neglecting that alternative splicing of transcripts from the same gene can give rise to protein isoforms that differ in both function and activity (Manuel et al. 2023).

In this text, we will concentrate on orthologs, since they are subject to the ortholog conjecture. However, the methods we describe here may also be applied to detect the diversification of all homologs. In the context of transferring functional gene annotations, for example, there may be multiple candidate genes in a target species, if gene duplications after speciation gave rise to in-paralogs/co-orthologs (Sonnhammer and Koonin 2002). Consequently, it has been a long-standing challenge to identify the “least diverged ortholog” to find the best fit for the transfer of functional annotations (Mi et al. 2010). Collecting evidence for the diversification of biochemical activity and/or functional context, as we describe it here, provides information beyond average sequence identity to help in the annotation of gene families with a history of gene duplication.

### Detecting Diversification of Function

The function of a gene is jointly defined by its biochemical activity and by the network of functional interactions it is embedded into, its functional context. Several databases provide information about these interaction networks, for example, the Gene Ontology (GO; “Biological Process”) (Aleksander et al. 2023), the pathway maps of KEGG (Kanehisa et al. 2023), or the collection of protein interactions stored in STRING (Szklarczyk et al. 2023). Additional types of functional interactions include transcriptional co-expression patterns (Raina et al. 2023) or physical protein–protein interactions (Alonso-López et al. 2019). Therefore, when transferring functional annotations between orthologs, it is essential to adopt a system-level perspective that considers the broader interaction context. This includes assessing whether orthologs of the associated interaction partners are present alongside the ortholog of interest. To distinguish true lineage-specific adaptations from background noise, such assessments should be conducted across phylogenetically diverse sets of taxa (Škunca and Dessimoz 2015).

Examples of where this approach has been pursued include the tracing of functional components of eukaryotic ribosomal biogenesis in the Archaea (Ebersberger et al. 2014; Birikmen et al. 2021), cilia-forming protein complexes (Nevers et al. 2017), or for identifying virulence-related gene cluster in a human pathogen (Djahanschiri et al. 2022). Note that evolutionary co-occurrence of genes has also been used in the reverse approach to predict the functional integration of genes (Pellegrini et al. 1999; Tabach et al. 2013; Linard et al. 2021).

Notably, cases where only one or a few components of a system are detected require careful interpretation. This pattern could either indicate that an ancestrally complex functional system has been simplified, or that the missing components are provided by symbiotic partners. For example, the milkweed bug (*Onchopeltus fasciatus*) lacks one gene in the biosynthesis pathway for an essential amino acid. This loss is complemented by the insect’s microbiome (Panfilio et al. 2019), leaving the functional context of the represented biosynthesis factors unaltered. In a different example, *Microsporidia* lack many ribosomal biogenesis factors that are otherwise almost ubiquitously present throughout the eukaryotes (Birikmen et al. 2021). Here, it is currently discussed that *Microsporidia* indeed have altered their ribosome biogenesis pathway with the consequence that individual ribosome biogenesis factors have modified their functional context (Melnikov et al. 2018). Other well-documented examples for the process of functional repurposing include key developmental regulators in insects (Niwa et al. 2010), mitosis control genes in yeasts (Frost et al. 2012), or animal orthologs of fungal plant cell wall degrading enzymes (Tran et al. 2024).

The examples presented thus far assume that the biochemical activity of an ortholog, if it is present, has not changed. However, Birikmen et al. (2021) provided strong evidence for a functional divergence of large ribosomal subunit maturation between eukaryotes and Archaea despite the shared presence of the corresponding biogenesis factors. This divergence is driven by different biochemical activities of the respective orthologs indicated by the fact that specific protein features that are relevant to the protein activity in eukaryotes are absent in the archaeal orthologs (Birikmen et al. 2021). This is only one example of many that highlight that the biochemical activity of a gene can diverge across lineages as a function of evolutionary time (Studer and Robinson-Rechavi 2009). This places the ultimate focus on whether two orthologs still exert the same ancestral activity, or whether one—or both—have experienced a change in biochemical activity.

### Diversification of Biochemical Activity

The equivalence of both functional context and biochemical activity serves as the null hypothesis when analyzing orthologs. What is now needed are approaches capable of generating results that rigorously challenge this assumption. Experimental characterization is the gold standard for inferring the biochemical activity of a protein (Rentzsch and Orengo 2009). However, in silico studies have contributed significantly to the understanding of what drives the diversification in the biochemical activity of two evolutionarily related proteins. For example, comparative analyses between members of the GH18 family of chitinases (Huang et al. 2012) and of the gel-forming mucins (Lang et al. 2007) revealed that rearrangements of protein domains account for changes in the activity of orthologs. Higher resolving analyses revealed that modifications within a domain can also have this effect (Alvarez-Carreño et al. 2016; Iruegas et al. 2023). In an even more fine-grained example, lineage-specific adaptations balancing enzyme stability and biochemical activity have been linked to a single amino acid residue in the bacterial ketosteroid isomerase family (Pinney et al. 2021).

Consequently, a multi-scale approach is necessary to comprehensively challenge the hypothesis of equivalent activity for orthologs. The foundation is provided by the fact that the orthology concept can be applied to any evolutionary entity, such as genes, exons, or introns, protein domains (Persson et al. 2019), or even individual nucleotide positions (Hughes and Friedman 2005). This enables us to incrementally increase the resolution of comparative analyses to identify evolutionary changes that may impact the biochemical activity performed by orthologs.

### Changes in Feature Architecture

The functional analysis of a protein typically involves the annotation of *features* in the amino acid sequence that are directly or indirectly related to the activity of the protein. Examples include structural protein domains (Waman et al. 2024), regions of conserved sequence (Blum et al. 2025), signal peptides and transmembrane domains (Krogh et al. 2001), and low-complexity regions (Ziemska-Legiecka et al. 2024). Alterations in protein feature architectures (FAs) have been associated with changes in protein activity (Lang et al. 2007; Huang et al. 2012; Iruegas et al. 2023). Therefore, the extent of FA divergence between orthologs can be used to challenge the null hypothesis of equivalent biochemical activity. High-quality annotation transfer efforts such as those by the GO Consortium using the manual curation tool PAINT (Gaudet et al. 2011), as well as automated procedures such as BlastKOala (Kanehisa et al. 2016) or KOFamKoala (Aramaki et al. 2020) use FA comparisons in the annotation process. Moreover, orthology databases such as InParanoid (Persson and Sonnhammer 2023), OMA (Altenhoff, Warwick Vesztrocy, et al. 2024), and OrthoDB (Kuznetsov et al. 2023) provide Pfam domain architectures as accessory information. However, comparing FAs with these approaches either requires visual inspection, which is hard to standardize and unsuited for large-scale comparative analyses, or they are restricted to proteins represented in certain databases. The development of efficient scoring functions of FA similarity (Koestler et al. 2010; Dosch et al. 2023) has opened the possibility to routinely consider FA similarity in large-scale comparative analyses (Birikmen et al. 2021; Djahanschiri et al. 2022; Tran et al. 2024). Importantly, FA-based comparisons have revealed changes in biochemical activity, even in highly conserved genes that would have been dismissed as uninformative in a purely gene presence–absence-based analysis (Iruegas et al. 2023). This underscores the power of a multi-level approach to uncover biologically meaningful divergence that would remain invisible at coarser levels of resolution.

FA dissimilarities suggest divergence in ortholog activities (Dosch et al. 2023), but again, the absence of such differences does not imply functional equivalence. When framed as a null hypothesis, failing to reject equivalence is inherently a weaker conclusion—it reflects insufficient evidence rather than confirmation. A main reason lies in the limited feature space used in FA analyses. The biological sequence landscape is vast, and much of it remains unexplored (Río et al. 2024). Although databases of structural domains and conserved regions are expanding (Waman et al. 2024; Blum et al. 2025), they remain incomplete. This constrains FA comparisons to the narrow subset of features represented in databases, with the consequence of them potentially underestimating functional divergence.

In addition, FA-based analyses must account for the construction of the underlying feature models. Databases such as Pfam (Mistry et al. 2021) represent features as probabilistic models (pHMMs). To avoid overfitting (i.e., the model fits well to the training data but generalizes poorly to non-observed instances of the modelled entity), data for training the models should be gathered to maximize sequence diversity. While this generalist approach facilitates the broad propagation of feature annotations across organisms, it has one major drawback. Homologous features that have diverged in their activity may still produce significant hits against the same model, thereby obscuring signals of functional divergence in FA comparisons (Iruegas et al. 2023). As a result, orthologs with apparently conserved FA may nonetheless differ in their biochemical activity. These limitations underscore the importance of integrating further, more fine-grained levels of resolution into comparative analyses to test even more comprehensively for the divergence of ortholog activity.

### Finding the Appropriate Level of Resolution

Methodologically, it is straightforward to move from FA comparisons to the highest resolution level: individual sequence positions. Numerous examples demonstrate that substituting a single or a few amino acids can alter the biochemical activity of orthologous proteins (Figueroa et al. 1988; Safi et al. 2008; Boyle et al. 2017). A broad array of software tools supports the alignment of homologous sequences, ranging from pairwise alignments to genome-scale comparisons (e.g.,Darling et al. 2010; Armstrong et al. 2020; Kirilenko et al. 2023), rendering the identification of sequence differences straightforward. With the advent of AlphaFold, orthologs can now also be examined at the level of predicted 3D protein structures (Jumper et al. 2021). As with sequence-level analyses, structural deviations may signal functional changes (e.g., Iruegas et al. 2023). However, both sequence and structural comparisons operate at a level of resolution, where additional contextual information is needed to distinguish meaningful activity-altering changes from background variation. In many cases, such intermediate layers of resolution may be specific to the research question at hand. Consequently, one element of the multi-scale approach is to identify appropriate layers of additional information. In the following section, we will give an example of such an additional layer of resolution in the context of the emergence of SARS-CoV-2 as a human pathogen.

### The Multi-level Perspective in Action

SARS-CoV-2 belongs to the subgenus *Sarbecovirus* within the *Coronaviridae* (Gorbalenya et al. 2020). Most coronaviruses, but also most sarbecorviruses, are associated with only mild respiratory symptoms (Weiss and Navas-Martin 2005). The few notable exceptions include SARS-CoV-1 and SARS-CoV-2—the causative agents of the 2002 SARS epidemic (Coleman and Frieman 2014) and the COVID-19 pandemic in 2020 (Zhou et al. 2020), respectively. Tracing the evolutionary adaptations that distinguish these two potent human pathogens from other coronaviruses is critical to improving our understanding of how novel threats to human health emerge.

**Fig. 2:**
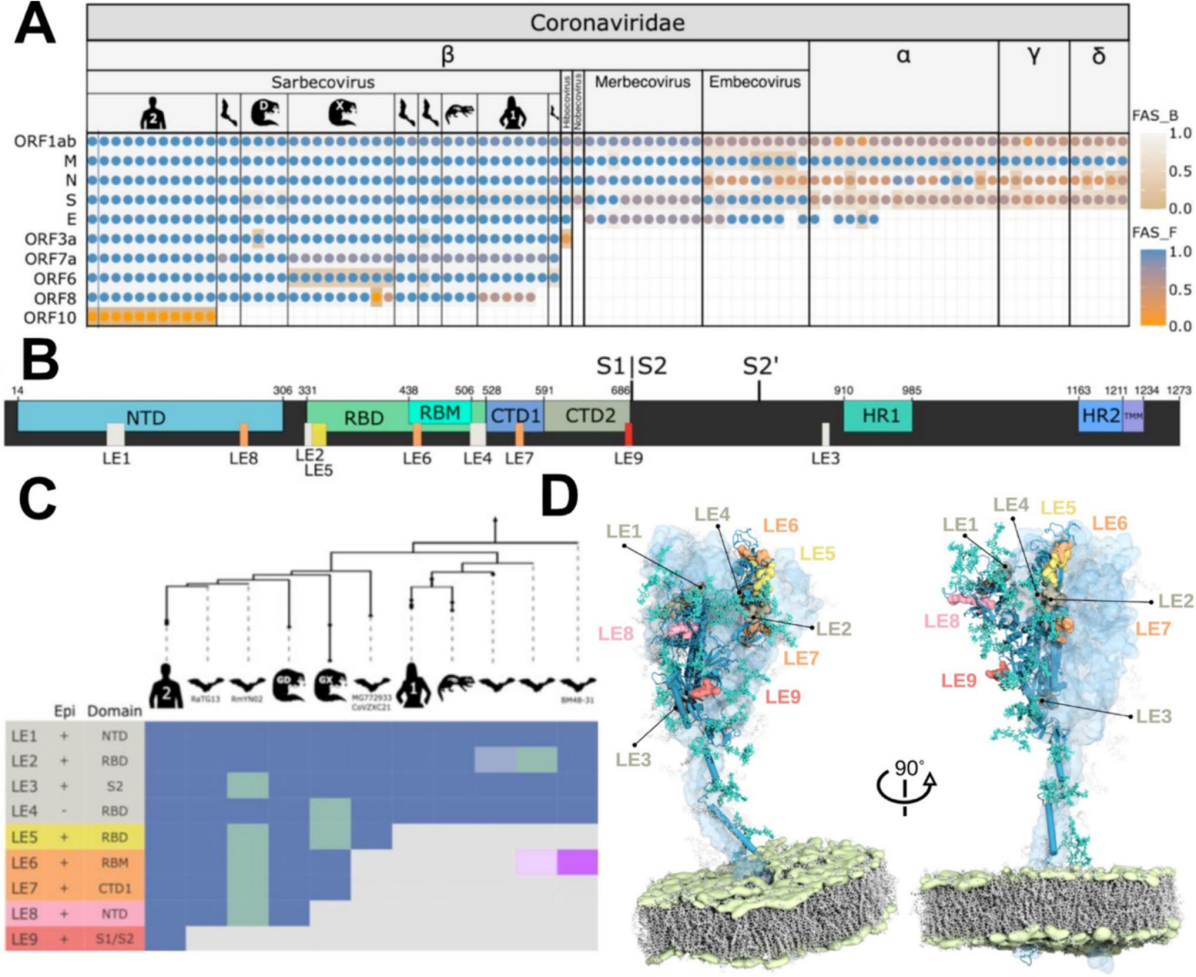
Multi-level perspective on the evolution of SARS-CoV-2. **A** Feature-aware phylogenetic profiles of SARS-CoV-2 proteins across the Coronaviridae. Sarbecoviral hosts are indicated by the pictograms. Human 1 and 2 represent SARS-CoV-1 and SARS-CoV-2, respectively. The Guangdong (Liu et al. 2019) and Guangxi (Lam et al. 2020) pangolin viruses are marked with ‘D’ and ‘X’, respectively. Taxa are ordered with increasing evolutionary distance to SARS-CoV-2 GCF_009858895.2, which was used as the seed species in this analysis. Dots indicate the detection of an ortholog in the respective species. Colors represent the feature architecture similarity between the seed protein and its ortholog using once the seed (dot color; FAS_F), and once the ortholog (cell color; FAS_B) as reference. *M* membrane glycoprotein, *N* nucleocapsid protein, *S* spike glycoprotein, *E* envelope protein. **B** Position of domains and linear epitopes in the spike. Represented are the N-terminal domain (NTD), receptor binding domain (RBD), receptor binding motif (RBM), C-terminal domain (CTD), furin cleavage site (S1|S2), second proteolytic cleavage site (S2’), heptad repeats (HR), and transmembrane region (TMM). Domains were assigned according to the annotations from (Cai et al. 2020). **C** Phylogenetic profile of predicted epitopes; the taxa are ordered according to (**A**). Epi: Overlap with experimentally verified epitopes (Shrock et al. 2020). Domain: The spike domain assignment for the LEs [c.f. (**B**)]. Epitope losses are indicated by a green cell color. The occurrence of LE6 in the early branching sarbecovirus lineage (purple cell color) is due to a convergent emergence of this epitope. LE2 and LE6 are present only in 1/3 of the bat viruses subsumed under the respective node in the tree, which is indicated by a lighter cell color. **D** Predicted linear epitopes annotated on a full-length model of a spike glycoprotein of SARS-CoV-2. The structural model (Sikora et al. 2021) represents the trimeric spike in a prefusion state embedded in a lipid bilayer (lipid tails marked gray and phosphate headgroups green). The epitopes in the surface representation are overlaid with the chain in the “up” conformation (blue cartoon). Realistic glycan coverage is shown as turquoise licorice. The remaining two chains in the “down” conformation are shown as light-blue transparent surfaces, with corresponding glycans transparent gray. The epitope color code follows (**B**)

Lineage-specific adaptations that distinguish sarbecoviruses from other members of the *Coronaviridae* family are detectable at both gene presence–absence level and through a more fine-grained FA analysis (Fig. 2A). However, neither level of resolution is sufficient to differentiate between the human–pathogenic sarbecoviruses and those that infect only animals. Thus, changes that turned SARS-CoV-2 into a potent human pathogen need to be sought on a different level. Soon after sequencing the first isolates of SARS-CoV-2, comparative genome analyses provided a comparison of sarbecoviral genomes including SARS-CoV-2 with a single-nucleotide resolution (Zhou et al. 2020). RaTG13, a virus isolated from a bat, was identified as the closest relative to SARS-CoV-2 with a genome-wide sequence similarity of 96.2%. A nucleotide-level resolution comparison between the two viral genomes should provide the most direct access to genomic changes underlying the virulence of SARS-CoV-2. However, genomic differences are roughly evenly distributed across the viral genomes. Although a slightly higher divergence in the gene encoding the viral spike protein was observed, other sarbecoviruses had also markedly diverged in this region, making it hard to pinpoint the causative change in the human pathogen (Zhou et al. 2020). In summary, comparative analyses on FA level lack sufficient resolution, whereas genome-wide analyses of single-nucleotide changes are overly fine-grained and yield an unfavorable signal-to-noise ratio. Consequently, an intermediate level of resolution must be found to reveal the protein changes in SARS-CoV-2 that enabled its shift from animal to human hosts. To further increase the signal-to-noise ratio, we narrowed the scope of this analysis to orthologs of the spike protein, since this protein mediates the first interaction between the virus and its host.

To add a layer of resolution that is intermediate between FA changes and nucleotide positions in the context of viral proteins, we traced the phylogenetic profiles of individual epitopes. Epitopes are short structural motifs recognized by the host immune system, shaping the immunogenic properties of viral proteins (Huang and Honda 2006). In silico prediction tools (Moutaftsi et al. 2006; Wang et al. 2008) identified nine linear epitopes (LE) in the SARS-CoV-2 spike protein (Fig. 2B). Subsequently, we traced the presence of these epitopes across the sarbecorivus diversity. Notably, the number of shared epitopes declines with increasing phylogenetic distance from SARS-CoV-2 (Fig. 2C), indicating progressive divergence. One epitope (LE9) appears unique to SARS-CoV-2, which strongly suggests that it arose after divergence from its closest known relative, RaTG13 (Fig. 2C). Interestingly, LE9 overlaps with the furin cleavage site—a region implicated in both immune evasion and enhanced host cell entry (Shang et al. 2020), making it a strong candidate for a change that affects the biochemical activity of the SARS-CoV-2 spike protein.

The initial epitope predictions did not incorporate 3D structural or glycosylation data (Watanabe et al. 2020), leaving it unresolved which epitopes are located on the surface of the protein. However, a subsequent addition of both layers of information (Sikora et al. 2021) to the multi-perspective comparison confirmed that LE9 is surface-exposed and accessible for host interactions (Fig. 2D). The importance of the LE9 epitope is meanwhile corroborated by mounting evidence which suggests that changes in the furin cleavage site were indeed key to the successful human adaptation of SARS-CoV-2 (Johnson et al. 2021).

In summary, the example of SARS-CoV-2 demonstrates that it is necessary to consider multiple, sometimes question-specific levels of resolution, such as the epitope landscapes of viral proteins, to trace signals of diversification in biochemical activity. Unfortunately, predicting the appropriate level of resolution a priori is challenging. This makes it necessary to rely on flexible frameworks for comparative analyses that allow us to integrate various layers of information on the fly.

## Conclusion and Outlook

Orthology and the conservation of biochemical activity, as well as functional context, are often assumed to be tightly interlinked. For closely related species, this simplified view may hold true in many cases. However, over deep evolutionary timescales, such assumptions become increasingly unreliable. It becomes essential to distinguish between orthologs that have retained their ancestral activity and functional context, and those that have diverged. Research communities focused on the evolution of specific proteins or pathways over long timescales are generally aware of this complexity and often rely on expert curation to avoid spurious annotation transfers. Yet, with the rise of biodiversity genomics and large-scale proteome analyses spanning hundreds or thousands of species, manual curation becomes infeasible, and the risk of inaccurate annotation transfer grows substantially.

To address this challenge, we argue that the equivalence of biochemical activity and functional context between orthologs should be treated as a null hypothesis—one that must be actively tested rather than assumed. Challenging this hypothesis requires multi-level comparative analyses: from assessing the co-occurrence of functional interaction partners to detecting subtle changes in sequence or structure that might affect protein activity. Importantly, shifting comparative analyses of diversifying activity to higher resolution layers comes with a trade-off: it substantially increases the number of data points that must be examined. This can quickly become unmanageable unless the scope of more fine-grained analysis is narrowed based on initial findings. Therefore, it remains best practice to begin with a high-level overview such as the presence–absence patterns of orthologs and to incrementally increase resolution in an iterative manner. As resolution increases, the number of candidate genes under investigation typically decreases. This tiered approach also supports the identification and curation of the data needed for a more detailed investigation of specific candidates.

The systematic integration of additional layers of information into comparative analyses necessitates metrics that capture diversification of biochemical activity, not just divergence of sequences or structures. Initial scoring schemes, such as those assessing protein feature architecture similarity (Dosch et al. 2023), represent early steps in this direction. AlphaMissense, a deep learning tool predicting the structural impact of amino acid substitutions (Cheng et al. 2023), marks another promising development toward the integration of structural data into comparative analyses. While it provides valuable insights into activity-altering missense mutations in the human genome, it was not designed to evaluate evolutionary divergence between orthologs. Dedicated frameworks that infer functional divergence over evolutionary timescales—particularly those incorporating protein structural data—are consequently still lacking. The development of such methods will help to routinely consider additional layers of information in multi-level comparative analyses of orthologs in the future.
